# Proteomics‐based identification of orchid-associated bacteria colonizing the *Epipactis albensis, E. helleborine* and *E. purpurata* (Orchidaceae, Neottieae)

**DOI:** 10.1016/j.sjbs.2021.04.002

**Published:** 2021-04-20

**Authors:** Anna Jakubska-Busse, Anna Kędziora, Gabriela Cieniuch, Agnieszka Korzeniowska-Kowal, Gabriela Bugla-Płoskońska

**Affiliations:** a University of Wroclaw, Faculty of Biological Sciences, Department of Botany, 50-328 Wroclaw, Poland; b University of Wroclaw, Faculty of Biological Sciences, Department of Microbiology, 51-148 Wroclaw, Poland; c Polish Collection of Microorganisms (PCM), Department of Immunology of Infectious Diseases, Hirszfeld Institute of Immunology and Experimental Therapy, Polish Academy of Sciences, Rudolfa Weigla 12, 53-114 Wroclaw, Poland

**Keywords:** *Epipactis*, Orchidaceae, Endophytic bacteria, Associative bacteria

## Abstract

Using proteomics-based identification by matrix-assisted laser desorption/ionization time-of-flight mass spectrometry (MALDI-TOF MS), we conducted the first analysis of the composition of endophytic bacteria isolated from different parts of selected *Epipactis* species, i.e. the buds, the inflorescences and the central part of the shoots, as well as the rhizomes. We identified aerobic and anaerobic bacteria, including such taxa as *Bacillus* spp., *Clostridium* spp., *Pseudomonas* spp. and *Stenotrophomonas* spp., which may be considered as promoting plant growth. Because most of the indicated bacteria genera belong to spore-producing taxa (spores allow bacterial symbionts to survive adverse conditions), we suggest that these bacteria species contribute to the adaptation of orchids to the environment. We found clear differences in the microbiome between investigated closely related taxa, i.e., *Epipactis albensis, E. helleborine, E. purpurata* and *E. purpurata* f. *chlorophylla*. Some of the analysed orchid species, i.e. *E. albensis* and *E. purpurata* co-occur in habitats, and their bacterial microbiomes differ from each other.

## 1 Introduction

The complex co-associations of plants with endophytic organisms, including bacteria, fungi, protists, nematodes and viruses have important roles in health of the plant, confer advantages including growth promotion, nutrient uptake, stress tolerance and resistance to pathogens (Trivedi et al., 2020). The concept that plants and the associated microbiota form a 'holobiont' has become popular and discussed (Vandenkoornhuyse et al., 2015). It should be mentioned that, the associations of microbiota with their host plants are varied and complex (Tadych et al., 2009). General structure of the bacterial and fungal communities depends on the plant compartment, environment, geographic location and host. The plant-associated microbiome is dynamic during lifecycle of the plant (Trivedi et al., 2020). Bacteria probably have a potential use/role as plant growth promoters, especially in acclimatizing seedlings obtained by micropropagation as well as nutrient uptake and pathogen resistance (Azizoglu, 2019; Dias et al., 2009; Trivedi et al., 2020) Beneficial microbiome protects the plant against pathogens by the production of antibiotics, lytic enzymes, volatiles and siderophores and can produce a range of enzymes that can detoxify reactive oxygen species (Vandenkoornhuyse et al., 2015). Moreover, plant-associated bacteria usually remain resistant to bacteriocines, so they might be a stable component of bacteria-plant symbiosis (Lee et al., 2016, Flores-Treviño et al., 2004, Liu et al., 2020). Edophytic bacteria and fungi, mainly yeast form a powerful consortium based on strong networks of coexistence and dependence (Villarreal-Soto et al., 2018).

The composition and role of orchid-associated bacteria (OAB) colonizing the underground tissues of terrestrial European orchids is relatively poorly understood. Tropical orchids have been scientifically well researched in this regard. Galdiano et al. (2011), based on partial sequencing of the 16S rRNA genes of bacteria cultures from root velamen of *Cattleya walkeriana*, identified four taxa of rhizobacteria, i.e. *Bacillus* sp., *Burkholderia* sp., *Enterobacter* sp. and *Curtobacterium* sp. These rhizobacteria can produce auxin, which favorably influences the growth *C. walkeriana* germinated in asymbiotic conditions and during the acclimatization process (Galdiano et al., 2011). Endophytic bacteria identified in the epiphytic orchid *Dendrobium moschatum* were recognized among the other genus: *Rhizobium* sp., *Microbacterium* sp., *Sphingomonas* sp., and *Mycobacterium* sp. They are responsible for plant growth promotion by producing indole acetic acid (IAA) and solubilizing inorganic phosphate (Malboodi et al., 2009). Additionally, it was proved that inoculation of *D. moschatum* seeds with *Sphingomonas* sp. and *Mycobacterium* sp. resulted in considerable enhancement of orchid seeds germination (Tsavkelova et al., 2007a, Tsavkelova et al., 2007b, Zhang and Song, 2012). Research conducted on Thai species of the genus *Cymbidium* has shown that it follows a seasonal pattern of abundance that differed between orchid genera, especially on the morphological level of the endophyte-infected tissue (Vendramin et al., 2010, Chutima et al., 2011). Identified species of bacteria that can promote plant growth of *Cymbidium* sp. orchids are the following: *Bacillus thuringiensis*, *Burkholderia cepacia*, *Burkholderia gladioli*, *Herbaspirillum frisingense*, *Pseudomonas stutzeri*, *Rhizobium cellulosilyticum*, *Rhizobium radiobacter*, and *Stenotrophomonas maltophilia* (Gontijo et al., 2018). According to the literature data, some strains of OAB were able to promote the symbiotic germination of *Cymbidium goeringii*, *Orchis militaris* and *Holcoglossum* species (Sun et al., 2009, Vendramin et al., 2010, Tan et al., 2012). Therefore, it seems that bacteria play an equally important role in the orchid life cycle as mycorrhizal fungi.

The *Epipactis* species are well known in terms of the mycobiota that inhabits it, e.g. Ogórek et al. (2020), however, there is no data on endophytic bacteria, the presence of which may be of key importance in their adaptation to the environment.

In this in this preliminary studies the following issues have been raised for the first time: (i) isolation and identification the orchid-associated bacterial (OAB) endophytes of *Epipactis* species including vegetative plant organs, (ii) a comparison of the OAB between the species of the genus *Epipactis* including the taxonomy and habitat of the tested orchid and (iii) an attempt to explain their importance in the biology of the studied *Epipactis* taxa based on the literature data.

## 2 Material and methods

### 2.1 Plant materials

**Fig. 1:**
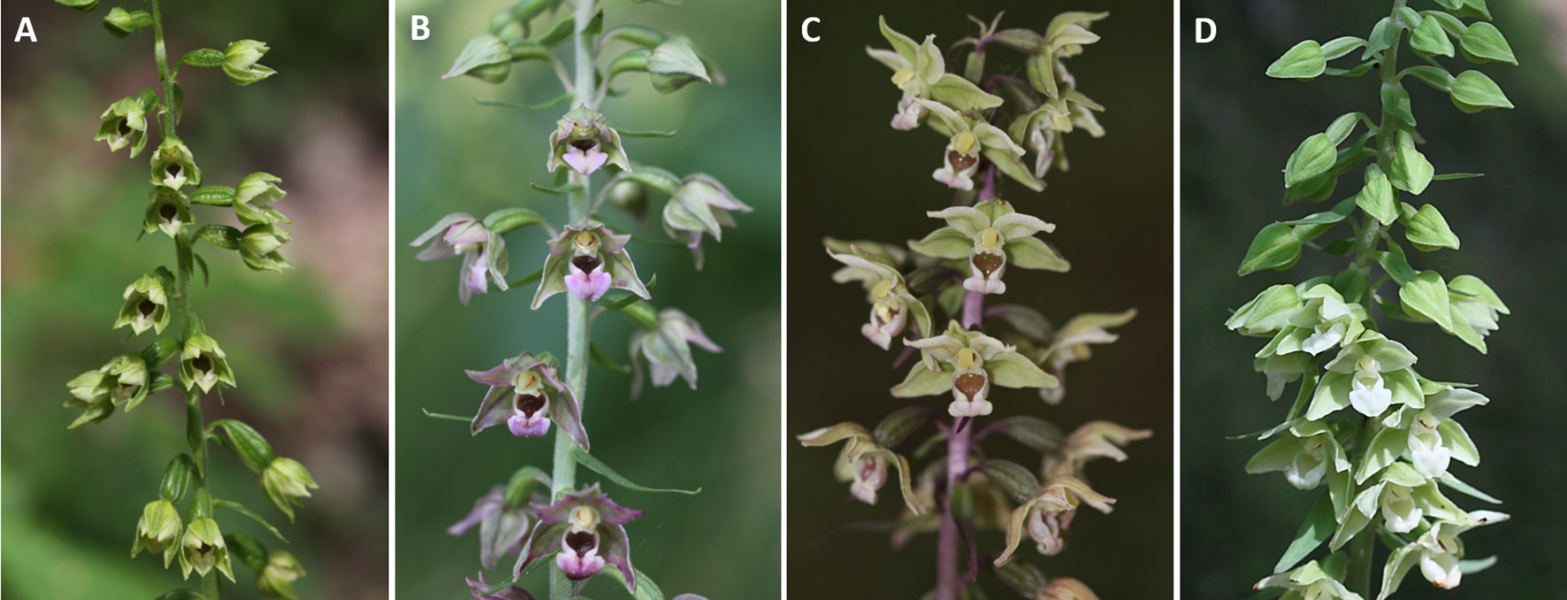
General habit of investigated orchid taxa. (A) *Epipactis albensis*, (B) *Epipactis helleborine*, (C) *Epipactis purpurata* and (D) *Epipactis purpurata* f. *chlorophylla*.

Plants belonging to three ecologically diverging species of the mixotrophic *Epipactis* genus, i.e. *Epipactis helleborine* (L.) Crantz, *E. albensis* Nováková et Rydlo, *E. purpurata* Sm. and rare intraspecific taxon *E. purpurata* Sm. f. *chlorophylla* (Seeland) P. Delforge were analyzed (Fig. 1). All the taxa

were identified using their morphological character on the basis of the literature data, e.g. Baumann et al., 2006, Delforge, 2006, Jakubska-Busse et al., 2017, Żołubak et al., 2017. Plant samples were collected from three plant parts, i.e. closed flower buds, stem, rhizome fragments and adventitious roots. Plant material was collected in the summer of 2020, from natural populations of *Epipactis purpurata, E. purpurata* f. *chlorophylla* and *E. albensis* growing in modified Central European oak-hornbeam forest, *Galio-Carpinetum* in the Nieszczyce (SW Poland), as well as *Epipactis helleborine* (L.) Crantz from Trestno (SW Poland, Wrocław County) growing in the regenerative forest and bush communities referring to the riparian habitat or riparian woodland classified into the *Salicetea purpureae* class. GPS (Global Positioning System) coordinates are available from the authors upon request. All species of the genus *Epipactis* are legally protected in Poland, and only a small number of genets - genetic individuals (9) have been authorized to conduct research in this project. In the studied population, we examined 30 ramets classified to different *Epipactis* species. Material sampling was done with permission no. WPN.6400.24.2020.MH from the Regional Directors for Environmental Protection.

### 2.2 Isolation of endophytic bacteria

The bacterial microflora was isolated in aerobic conditions using the following media: Nutrient Broth (BioMaxima, Lublin, Poland) and Enriched LAB-AGAR (BioMaxima, Lublin, Poland) and in anaerobic conditions using the following media: Schaedler Broth (BioMaxima, Lublin, Poland) and Schaedler Anaerobe LAB-AGAR™ + 5% SB (Biomerieux, France). Incubation in aerobic conditions of the material was carried out at 30℃ for 24 h, while incubation in anaerobic conditions was carried out at 30℃ for 72 h. The flowers, leaves, stems, rhizomes and shoots were cleaned by rinsing in 70% ethanol and then rinsing twice with milliQ water. The plant material after crushing was introduced into 5 ml of Nutrient Broth medium and 10 ml of Schaedler Broth medium and left for incubation. Swabs collected from inside of the orchids flower orchids were introduced into liquid media and incubated. The soil was suspended in 20 ml of sterile milliQ water and vortexed until a homogeneous solution was obtained. Then 1 ml of the soil solution was introduced into the liquid medium of Nutrient Broth and Schaedler Broth and incubated. After incubation, the material was plated on solid Enriched LAB-AGAR (30℃/24 h) and Schaedler Anaerobe LAB-AGAR ™ + 5% SB (30℃/72 h) (Faria et al., 2019).

### 2.3 Identification of bacterial isolates

All obtained bacterial isolates were analyzed using MALDI (Matrix-Assisted Laser Desorption Ionization Time-of-Flight) Biotyper method as described before (Książczyk, et al. 2016). Briefly, ribosomal proteins were extracted using 70% formic acid and acetonitrile method. Next, each sample was spotted on a 384 ground steel MALDI target plate. After drying, the applied sample, an equal volume of matrix (α-cyano-4-hydroxy-cinnamic acid (HCCA)) was spotted onto the spot. Then, the plate was left at room temperature for about 15 min to dry. Mass spectra of the extracted proteins was measured using the mass spectrometer MALDI-TOF ultrafleXtreme (Bruker Daltonics GmbH, Bremen, Germany). To identify bacterial mass spectra, Biotyper 3.1 software and database containing 6904 entries were used (Bruker Daltonics GmbH, Bremen, Germany). Based on ‘Bruker Daltonik MALDI Biotyper Classification Results’ protocol the following score criteria were applied to identification of bacteria: 2.300–3.000 - highly species identification, 2.000–2.299 - probable species identification, 1.700–1.999 - probable genus identification, 0.000–1.699 - not reliable identification.

## 3 Results

### 3.1 Orchid associated bacteria

**Fig. 2:**
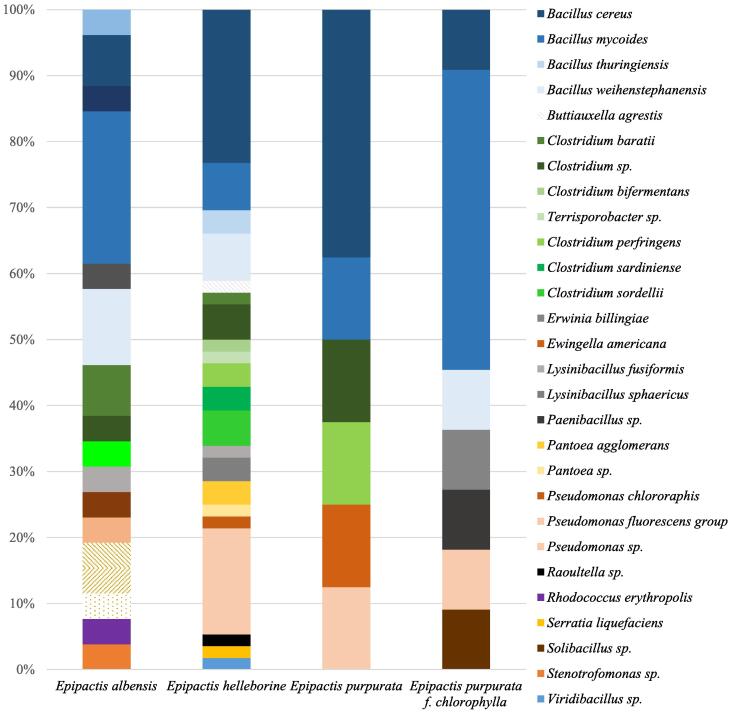
Percentage of each bacterial species contributing to the totals for the *Epipactis albensis, E. helleborine, E. purpurata* and *E. purpurata* f. *chlorophylla.*

The used methods of orchid-associated bacteria (OAB) under aerobic and anaerobic conditions allowed us to obtain a total of 192 isolates. The MALDI Biotyper method assigned 103 isolates to species level. Analysis of the orchid associated microorganisms showed the presence of 20 different bacterial species within *Epipactis albensis, Epipactis helleborine, Epipactis purpurata* and *Epipactis purpurata* f. *chlorophylla*. The list of general identified bacteria from *Epipactis* species is presented in Table 1. Because the endophytic bacteria were isolated from different part of the orchid (ground and underground), a summary of the obtained data is presented in detail in Table 2. Percentage representations of each bacterial species contributing to the total isolates from every of *Epipactis* are summarized in Fig. 2. Moreover roots in some single genets were found - *Epipactis helleborine* ramet 3 and *Epipactis purpurata* f*. chlorophylla* and bacteria strains were isolated with differences between both individuals. In case of *Epipactis helleborine* ramet 3 the following bacteria strains were indicated: *Buttiauxella agrestis, Pseudomonas putida* and *Raoultella ornithinolytica*, while *Bacillus mycoides* and *Bacillus cereus* were selected from *Epipactis purpurata* f*. chlorophylla.*

**Table 1:** List of orchid-associated bacteria (OAB) species isolated from *Epipactis* spp.

Isolated bacteria species	*Epipactis albensis*	*Epipactis helleborine*	*Epipactis purpurata*	*Epipactis purpurata* f. *chlorophylla*
*Bacillus* sp.	*+*	+	+	*+*
*Bacillus cereus*	*+*	+	+	*+*
*Bacillus mycoides*	+	+	+	+
*Bacillus thuringiensis*		+	–	–
*Bacillus weihenstephanensis*	+	+	–	+
*Buttiauxella agrestis*	–	+	–	–
*Clostridium baratii*	+	+	–	–
*Clostridium bifermentans*	–	+	–	–
*Terrisporobacter* sp.	+	+	–	–
*Clostridium* sp.	+	+	+	–
*Clostridium perfringens*	–	+	+	–
*Clostridium sardiniense*	–	+	–	–
*Clostridium sordellii*	–	+	–	–
*Erwinia billingiae*	–	–	–	+
*Ewingella americana*	–	–	+	–
*Lysinibacillus* sp.	+	–	+	+
*Lysinibacillus fusiformis*	+	+	–	–
*Lysinibacillus sphaericus*	*–*	+	*–*	*–*
*Paenibacillus* sp.	*–*	–	*–*	*+*
*Paenibacillus amylolyticus*	+	–	–	–
*Pantoea* sp.	–	–	–	+
*Pantoea agglomerans*	–	+	–	–
*Pseudomonas* sp.	–	+	–	–
*Pseudomonas chlororaphis*	–	+	–	–
*Pseudomonas extremorientalis*	–	+	–	–
*Pseudomonas fluorescens* group	–	–	+	–
*Pseudomonas grimontii*	–	+	–	–
*Raoultella ornithinolytica*	*–*	+	–	*–*
*Rhodococcus* sp.	*+*	–	–	*–*
*Rhodococcus erythropolis*	+	–	–	–
*Serratia liquefaciens*	–	+	–	–
*Solibacillus* sp.	–	–	–	+
*Stenotrofomonas* sp.	+	–	–	–
*Viridibacillus* sp.	–	+	–	–

**Table 2:** Diversity of orchid-associated bacteria (OAB) isolated from *Epipactis albensis*, *Epipactis helleborine* and *Epipactis purpurata* (n/a – not applicable, lacs of the source, X – no isolates founded).

	*Epipactis albensis*			*Epipactis helleborine*				*Epipactis purpurata*	*Epipactis purpurata* f*. chlorophylla*
Source	Ramet 1	Ramet 2	Ramet 3	Ramet 1	Ramet 2	Ramet 3	Ramet 4		
**Flower buds**	*Rhodococcus* sp.	*Bacillus mycoides*	X	X	X	X	*Pantoea ananatis*	X	*Bacillus* sp.
									*Erwinia billingiae*
**Closed flowers**	*Bacillus mycoides*	*Bacillus mycoides*	*Bacillus* sp.	*Bacillus* sp.	*Bacillus thuringiensis*	*Bacillus cereus*	*Staphylococcus warneri*	*Bacillus* sp.	*Bacillus mycoides*
	*Rhodococcus erythropolis*			*Bacillus thuringiensis*	*Bacillus cereus*	*Bacillus weihenstephanensis*	*Staphylococcus* sp.	*Clostridium* sp.	*Lysinibacillus* sp.
				*Bacillus cereus*	*Clostridium sordellii*	*Bacillus* sp.			*Paenibacillus* sp.
				*Clostridium sardiniense* *Clostridium* sp.	*Lysinibacillus sphaericus*				
				*Viridibacillus* sp.					
**Inflorescence**	*Bacillus* sp.	*Bacillus weihenstephanensis*	*Bacillus* sp.	X	*Bacillus weihenstephanensis*	*Bacillus cereus*	*Bacillus* sp.	*Bacillus cereus*	*Bacillus* sp.
	*Stenotrofomonas* sp.	*Bacillus amyloliticus* *Bacillus* sp.						*Lysinibacillus fusiformis*	*Bacillus mycoides*
							
**Middle part of the shoot (at the height of the leaves)**	X	*Bacillus mycoides*	*Bacillus* sp.	*Bacillus* sp.	*Bacillus* sp.	*Bacillus cereus*	*Bacillus cereus*	*Clostridium perfringens*	*Bacillus* sp.
		*Bacillus* sp.	*Lysinibacillus* sp.			*Bacillus* sp.	*Bacillus* sp.	***Ewingella americana***	*Bacillus mycoides*
									*Pseudomonas* sp.
				*Terrisporobacter* sp*.*	*Lysinibacillus* sp.	*Clostridium perfringens*			
							*Staphylococcus warneri*		
**Leaves**	*Bacillus mycoides*	*Bacillus weihenstephanensis*	*Bacillus* sp.	*Bacillus cereus*	*Bacillus mycoides*	*Bacillus* sp.	*Bacillus* sp.	*Bacillus cereus*	*Bacillus weihenstephanensis*
	*Clostridium baratii*	*Bacillus mycoides*	*Bacillus cereus*	*Clostridium sordellii*	*Bacillus cereus*	*Clostridium sordellii*	*Bacillus cereus*		*Lysinibacillus* sp.
	*Lysinibacillus* sp.	*Lysinibacillus* sp.		*Pseudomonas* sp.	*Clostridium perfringens*	*Clostridium baratii*	*Bacillus mycoides*		*Bacillus* sp.
				*Pseudomonas chlororaphis*	*Clostridium bifermentans*		*Bacillus weihenstephanensis*		
				*Pantoea agglomerans*			*Pseudomonas* sp.		
							*Pantoea agglomerans*		
**Rhizome**	*Bacillus cereus*	X	*Bacillus weihenstephanensis*	*Bacillus weihenstephanensis*	*Bacillus cereus*	*Bacillus* sp.	*Bacillus cereus*	*Bacillus cereus*	*Bacillus mycoides*
	*Clostridium baratii*		*Clostridium* sp. *Bacillus* sp.	*Bacillus* sp.	*Bacillus* sp.	*Bacillus cereus*	*Bacillus* sp.	*Bacillus mycoides*	*Bacillus* sp.
	*Lysinibacillus fusiformis*			*Pseudomonas* sp.	*Lysinibacillus sphaericus*	*Bacillus mycoides*	*Bacillus mycoides*	*Bacillus* sp.	*Solibacillus* sp*.*
					*Serratia liquefaciens*	*Clostridium* sp.	*Clostridium sardiniense*	*Pseudomonas fluorescens group*	
						*Pseudomonas* sp.	*Clostridium* sp.		
							*Pseudomonas* sp.

Among the identified bacterial taxa in every tested orchid species, the following taxa were identified: *Bacillus mycoides*, *B. weihenstephanensis* and *Lysinibacillus fusiformis*. We found that the studied specimens of two related taxa, i.e. *Epipactis purpurata* and intraspecific taxon *E. purpurata* f. *chlorophylla*, despite their very close taxonomic relationship, differ in the composition of endophytic bacteria. In the case of the tested *Epipactis* spp., dominating groups of endophytic bacteria usually belong to spore forming genus such as *Bacillus* and *Clostridium,* and non-spore forming *Pseudomonas* (Table 3). All of the mentioned genera have good adaptability to the different, variable and extreme environmental conditions. This correlates with the high tolerance of environmental conditions among the tested orchids and may explain the varieties of strains within the *Epipactis* species. It seems there is no bacterial species specificity within the *Epipactis* species. We found some differences in the microbiome between closely related taxa, i.e. *E. purpurata* and E. *purpurata* f. *chlorophylla*. Unfortunately, due to the small number of studied individuals, we treat these results as preliminary. To attempt to explain the significance of bacteria in the biology of the studied *Epipactis* taxa based on the literature data, we analysed the selected biological function of identified bacteria species (Table 3).

**Table 3:** Selected biological function of orchid-associated bacteria (OAB).

Orchid endophytes taxa	Selected biological function	References
*Bacillus* sp. *(B. cereus,* *B. licheniformis,* *B. mycoides,* *B. pseudomycoides,* *B. thuringiensis,* *B. weihenstephanensis)*	in general: aerobic Gram-positive bacteria, spore-forming, widely spread, causing: majority of them recognized as plant growth promoting by biofilm formation and antifungal and antipathogenic protection (biocontrol), mammal or other animal pathogen), isolated from soil and growing plants, psychrotolerant, nitrogen fixation activity, biostimulation and biofertilizer features, successful endophyte in many plants, plant growth promoting by suppressing diseases, antagonistic effects on phytopathogen, rhizoid colony morphology	Hollensteiner et al., 2017, Nel et al., 2019, Arnesen et al., 2008, Yi et al., 2018, Azizoglu et al., 2019
*Buttiauxella agrestis*	Gram-negative, isolated from environment and animal: surface water, soil	Jothikumar et al., 2014
*Clostridium* sp. (*Clostridium baratii, Clostridium bifermentans, Clostridium perfringens, Clostridium sordellii)*	in general: anaerobic, Gram-positive, spore forming bacteria, some plant associated, widely spread in the environment, plant associated microbes with different tissue, metabolic activity associated with fermentation, yeast and bacteria interactions favoured the survival of *C. bifermentans* and *E. cloacae* at the acidic pH typical of fermented cucumbers	Flythea et al., 2004; Neuhaus et al., 2015, Franco and Pérez-Diaz, 2012; Sarria-Guzmán et al., 2016, Kazanavičiūtė et al., 2018
*Terrisporobacter* sp.	relatively little-known pathogenic potential, often in conjunction with other pathogens	Cheng et al., 2016
*Clostridium sporogenes*	gut symbiont, generates aromatic amino acid metabolites such as tryptophan, phenylalanine and tyrosine, ferments amino acids and produces large amounts of acetate and butyrate with smaller amounts of isobutyrate, isovalerate, propionate, valerate, isocaproate, lactate and succinat*,* some strains produce butanol and ethanol if glucose is provided as an energy source	Flythea et al., 2004
*Erwinia billingiae*	Gram-negative, usually pathogenic to plants, human infections by *Erwinia*-like microorganisms are rarely described	Prod'homme et al., 2017
*Ewingella americana*	Gram-negative, cosmopolitan bacterial pathogen that has been isolated from many hosts, i.e. mushrooms, plant growth promoting, the strains encoded a set of common genes for type secretion, virulence effectors, CAZymes, and toxins required for pathogenicity in all hosts, antibiotic resistance, pigments to suppress or evade host defense responses, ability for adaptation to different environmental conditions, including temperature, oxidation, and nutrients (host adaptation strategies of *Ewingella*), and they also contribute to the development of effective control strategies	Liu et al., 2020
*Lysinibacillus fusiformis*	Gram-positive, isolated from factory wastewater and farming soil, can generate endospores, causes bacteremia, tropical ulcers	Sulaiman et al., 2018
*Lysinibacillus sphaericus*	Gram-positive, insect pathogen, especially for mosquitoes, lethal effects on eggs of the nematode *Trichostrongus colubriformis* and effects on the grass shrimp *Palaemonetes pugio,* produce bacteriocins and toxins against some cockroach and mosquitocidal	Berry, 2012
*Paenibacillus amylolyticus*	Gram-positive, spore forming, aerobic or facultatively anaerobic bacteria, isolated from: soil, fresh and salt water, sewage, sediments, caves, humus, compost, rhizosphere, food, plants, insect larvae and clinical sample, rhizosphere soil of peanut, produce of siderophore, promote the iron absorption of plant in calcareous soil, thus promoting plant growth	Liu et al., 2017, Sáez-Nieto et al., 2017
*Pantoea agglomerans*	Gram-negative, plant-associated bacteria, occur commonly, usually as symbionts, in insects and other arthropods, occur in plants as an epi- or endophytic symbiont, often as mutualist, cause of diseases in a range of cultivable plants, such as cotton, sweet onion, rice, maize, sorghum, bamboo, walnut, an ornamental plant called Chinese taro (*Alocasia cucullata*), and a grass called onion couch (*Arrhenatherum elatius*)	Dutkiewicz et al., 2016
*Pseudomonas fluorescens* group	Gram-negative, isolated from agricultural soil, well adapted to grow in the rhizosphere, rhizobacterium, biocontrol agent and promote plant growth ability, produce a wide spectrum of bioactive metabolites, i.e. antibiotics, siderophores, volatiles, and growth-promoting substances, responsible for the natural suppressiveness of some soilborne pathogen	David et al., 2018
*Pseudomonas* sp. *(Pseudomonas chlororaphis, Pseudomonas koreensis, Pseudomonas putida)*	widely spread, plant growth promoting (responsible for biofertilization, phytostimulation, and biocontrol), associated with soil and plant roots, biological control against phytopathogenic fungi, plant-colonizing and antagonistic activities against soil-borne plant pathogen, presence of different antimicrobial and insecticidal compounds, cyclic peptides, siderophores, bacteriocins, molecules involved in beneficial plant-bacteria interactions, not capable of forming spores, antagonistic to plant pathogenic fungi of the genera *Fusarium, Bipolaris* and *Alternaria,* an abundant microbe in the soil close to the roots (rhizosphere) of plants, plant growth promotion in nitrogen uptake, phosphorous solubilization, production of phytohormones, volatile compounds, able to colonize and persist in root environments of different plants, biocontrol agent, induces plant systemic response, protecting the plant host against pathogen infection and proliferation, used in several rhizoremediation projects for the elimination of contaminants in soil	Arrebola et al., 2019, Ivanova et al., 2002, Sawada et al., 2019, Gomesa et al., 2017, Sawada et al., 2019, Molina et al., 2020
*Rhodococcus erythropolis*	Gram-positive, isolated from seawater, alpine soil or coastal sediments from the Arctic to the Antarctic, biocontrol agent isolated from potato; inhibit bacterial pathogen such as A*. tumefaciens, Ralstonia solanacearum, Pseudomonas syringae* and *Erwinia amylovora*, causes bloodstream infection in humans	Baba et al., 2009, Latour et al., 2013
*Serratia liquefaciens*	Gram-negative, inhibition of the growth of pathogenic bacteria by the production of heliotropin, antifungal properties thanks chitinases enzyme production, plant growth promoting bacteria	Kalbe et al., 1996, Cieniuch et al., 2019
*Solibacillus* sp.	Gram-positive, round endospore-forming bacterium, isolated from a forest soil near Braunschweig, Lower Saxony, Germany, spore surface showed a cauliflower-like fine structure, contains lysine in its cell wall, plant protective bacteria	Rheims et al., 1999, Lee et al., 2020
*Stenotrofomonas* sp.	Gram-negative, responsible for nosocomial infections in immunocompromised patients, high drug resistance bacteria, virulence factors of *S. maltophilia* include extracellular enzymes, lipopolysaccharides, fimbriae, adhesins, flagella, and biofilm	Flores-Treviño et al., 2004; Sesatty and Garza-González, 2019

We analyzed not only parts of the plant, but also the soil from the close site of the orchid (Table 4). The soil’s microflora of the all studied *Epipactis* spp. contain bacteria belonging to the following genera: *Achromobacter, Acinetobacter, Bacillus, Clostridium, Citrobacter, Escherichia, Hafnia, Kluyvera, Lactococcus, Lysinibacillus, Pseudomonas, Raoultella, Serratia* and *Stenotrophomonas* (identified bacteria species divided into each *Epipactis* species was summarized in Table 4. In total, the different bacteria species were endophytes belonging to the following genera: *Achromobacter, Acinetobacter, Citrobacter, Escherichia, Hafnia* and *Raoultella*.

**Table 4:** Orchid-associated microbiome isolated from soil, presented in selected examples.

*Epipactis albensis*			*Epipactis helleborine*				*Epipactis purpurata*	*Epipactis pur*purata f. *chloroplylla*
Ramet 1	Ramet 2	Ramet 3	Ramet 1	Ramet 2	Ramet 3	Ramet 4		
*Bacillus mycoides*	*Acinetobacter* sp*.*	*Bacillus mycoides*	*Raoultella planticola*	*Pseudomonas koreensis*	*Buttiauxella* sp*.*	*Lysinibacillus fusiformis*	*Lysinibacillus* sp*.*	*Bacillus* sp*.*
*Lactococcus* sp.	*Bacillus* sp*.*	*Achromobacter xylosoxidans*	*Bacillus cereus*	*Clostridium sardiniense*	*Acinetobacter* sp*.*	*Achromobacter piechaudii*	*Bacillus mycoides*	*Citrobacter* sp*.*
*Lysinibacillus* sp*.*	*Hafnia alvei*	*Lactococcus* sp*.*	*Hafnia alvei*	*Clostridium* sp*.*	*Clostridium butyricum*	*Clostridium sporogenes*	*Clostridium baratii*	*Serratia liquefaciens*
*Serratia liquefaciens*	*Lactococcus lactis*	*Serratia liquefaciens*	*Lactococcus garvieae*	*Serratia grimesii*	*Escherichia coli*	*Escherichia coli*	*Serratia liquefaciens*	*Stenotrofomonas* sp*.*
*Stenotrofomonas maltophilia*	*Pseudomonas* sp*.*				*Kluyvera cryocrescens*	*Serratia* sp*.*		
	*Serratia liquefaciens*				*Lactococcus lactis*			

A very interesting and unexpected result of our research was the finding that the soil microbiome differs from that of a specific ramets growing in the studied substrate. The co-occurrence of two plant species, i.e. *Epipactis purpurata* and *E. albensis* in close proximity does not confirm their common requirements for the presence of specific bacteria in the soil.

## 4 Discussion

The orchids classified to *Epipactis* genus are rather difficult to cultivate *in vitro* (Kunakhonnuruk et al., 2018), probably for this reason, no data is available on the bacterial microbiome and its potential contribution in the biology of these orchids.

The results of our research, which we treat as preliminary to a further scientific project, turned out to be very interesting for a number of reasons. Firstly, we found that related orchid taxa, often co-occurring in habitats, i.e. *Epipactis albensis*, *E. helleborine*, *E. purpurata* and *E. purpurata* f. *chlorophylla*, differ in their microbiomes. These findings are surprisingly different from the results of our previous work on mycological evaluation of *Epipactis helleborine* and *E. purpurata* (Ogórek et al., 2020), where we showed that these two analyzed ecologically diverging *Epipactis* species, although growing in diverse habitats, did not differ significantly in terms of the composition of natural mycobiota (Ogórek et al., 2020).

Endophytes are defined as an important group of endosymbiotic microorganisms widespread among plants that colonize the intercellular and intracellular spaces of all known plant organs but do not cause any plant diseases or significant morphological changes (Miliute et al., 2015). This group also has been targeted as a valuable source of bioactive compounds and secondary metabolites important in the plant life cycle. Unfortunately, the species composition of endophytes inhabiting orchids and their biological role are very poorly understood.

Some of the bacterial strains, the genera *Bacillus* and *Pseudomonas* we isolated from *Epipactis* orchids, were previously found in the underground roots of *Calanthe vestita* var. *rubro-oculata* (Tsavkelova et al., 2001). These West Australian orchids and the genus *Epipactis* are classified in the same subfamily *Epidendroidae.* It is difficult to validate what role these bacteria can play in the biology of the *Epipactis* orchids without thorough research, but it is possible that they also support plant growth and/or plant development. Interestingly, we found these bacteria in various parts of the studied plants, i.e. in shoots, leaves and flower buds. Trivedi et al. (2020) reviewed that most of the endophytic bacteria belong to Proteobacteria but in case of our study significant number of isolates belong to Firmicutes (such as *Bacillus* and *Clostridium* genus).

Also, according to the literature, infection of *Cattleya loddigesii* with *Paenibacillus macerans*, orchid endophytic auxin-producing bacteria promoted seedling growth during the acclimatization process (Faria et al., 2013). Interestingly, in our research we identified related species, i.e. *Paenibacillus illinoisensis* as infected *E. purpurata* f. *chlorophylla*. It is possible that there is an association between *Paenibacillus* species and mycorrhizal fungi and the roots are the main penetration pathway for endophytic microorganisms (Faria et al., 2013).

Some bacterial genera identified in *Epipactis* orchids isolated during this work have been known for nitrogen or phosphor uptake in plants, e.g. *Bacillus mycoides* (Yi et al., 2018), *Pseudomonas putida* (Molina et al., 2020), *P. koreensis* (Gomesa et al., 2017, Sawada et al., 2019). This fact supports the hypothesis that they might be an important factor in *Epipactis* species growth promotion.

According to the literature, some soil bacteria identified in orchids, such as *Pseudomonas*, may promote the development of vesicular–arbuscular mycorrhizas (Azcon-Aguilar and Barea, 1985, Tsavkelova et al., 2001). It is well known that inoculation of the orchid seeds with an *Azotobacter* and the root-nodule bacterium *Bacillus radicicola* promote their germination (Knudson, 1922; Tsavkelova et al., 2001). The germination of orchid seeds is also enhanced by their bacterial infection by the genera *Pseudomonas*, *Bacillus*, *Arthrobacter* and *Xanthomonas* (Wilkinson et al., 1989, Wilkinson et al., 1994, Tsavkelova et al., 2001).

Moreover, the plant-associated bacteria species including *Bacillus* species (like *Lysinibacillus sphaericus, Bacillus amyloliquefaciens B. cereus*, *B. mycoides* and *B. thuringiensis)* or *Pseudomonas* species (the most frequently a successful endophyte in many plants) promote their development via indirect control of phytopathogenic fungi growth (Hollensteiner et al., 2017, Nel et al., 2019, Table 3). Bacteria impact plants via different modes of action, such as ROS (Reactive Oxygen Species) production, fermentation product, enzymatic lysis of the structure components, presence of different antimicrobial and insecticidal compounds, cyclic peptides, siderophores, bacteriocins, molecules involved in beneficial plant-bacteria interactions or the up-regulation of the expression of genes (Baba et al., 2009, Liu et al., 2020, Latour et al., 2013, David et al., 2018, Yi et al., 2018, Arrebola et al., 2019, Gautam et al., 2019, Liu et al., 2019, Molina et al., 2020). Moreover, Liu et al. (2019) observed that *Bacillus amyloliquefaciens* could inhibit mycelial growth, the germination of the cysts and the swimming of the motile zoospores of *Phytophtora sojae*.

The other strains isolated by us belonging to the genus *Pseudomonas* (such as *Pseudomonas orientalis, P. koreensis, P. chlororaphis*) have been known for their antagonistic activity, especially in the rhizosphere, but also in the apple flower against sol borne plant pathogens (both fungi and bacteria) (Gomesa et al., 2017, Arrebola et al., 2019, Sawada et al., 2019).

It is possible that the ability to form biofilm within isolated bacteria genus (e.g. *Bacillus, Pseudomonas*) plays an important role in orchid growth promotion because of the protection of the vegetative cells against some the pathogenic strains (Yi et al., 2018).

Furthermore, it is interesting that some plant pathogens, considered previously as symbiotic bacteria, were recognized. We identified bacteria genera (including *Erwinia*, *Pseudomonas*, *Bacillus* and *Clostridium*) that may cause soft rots in living plant tissue as a consequence of the presence of a strong viral factor (Prod'homme et al., 2017, Liu et al., 2020), but the analyzed orchid did not show any disease symptoms.

Similarly, in our previous research on fungal communities of *Epipactis helleborine* and *E. purpurata*, we found that the plants were infected by three species of the genus *Fusarium* (*F. oxysporum*, *F. sporotrichioides* and *F. tricinctum*) (Ogórek et al., 2020). These fungi are recognized as pathogenic, but they may also have other functions in ecosystems. Interestingly, we also found that the presence in Helleborines of some others species of fungi, especially *Alternaria tenuissima*, *Epicoccum nigrum*, *Penicillium biourgeianum* and *Trichoderma viride*, which could be effective against both fungal and bacterial pathogens (Ogórek et al., 2020). Similarly, we did not observe any disease symptoms typical of infections with pathogenic fungi in the analyzed plants.

There are a lot of arguments proving that we should not marginalize the importance of the coexistence of bacteria and fungi in Helleborines, because these microorganisms most likely play an important role in the process of adaptation of orchids to a changing environment.

In the next planned research project, we would like to experimentally test the influence of the identified bacteria and seed germination of the tested orchid taxa of the *Epipactis* genus.

## 5 Conclusions

Analysis of the orchid-associated bacteria (OAB) showed the presence of 35 different bacterial species within *Epipactis albensis*, *Epipactis helleborine*, *Epipactis purpurata* and *Epipactis purpurata* f. *chlorophylla*. Most of the isolated OAB belong to spore-forming, Gram-positive bacteria (*Bacillus* and *Clostridium*). Moreover, a numerous group was represented by *Pseudomonas* species. Isolated bacterial endophytes are considered as growth-promoting factor and may be significant in plant growth and development. We indicated diversity of the bacterial microbiome between plants, that grew in different types of habitats. Analysis of the OAB isolated from the soil in which these ramets grew also confirmed the differences.

In our opinion, the presence of endophytic bacteria, especially classified to the group of prototrophic organisms, can stimulate the growth and development of *Epipactis* orchids, especially those plants that grow on nutrient-poor soils. This is of particular importance in the adaptation of plants to the new environmental conditions and in the process of colonizing new habitats and territories.

## Funding

Publication of this article in open access was financially supported by the Excellence Initiative - Research University (IDUB) programme for the University of Wroclaw.

## Declaration of Competing Interest

The authors declare that they have no known competing financial interests or personal relationships that could have appeared to influence the work reported in this paper.
